# Fabrication of Pneumatic Microvalve for Tall Microchannel Using Inclined Lithography

**DOI:** 10.3390/mi7120224

**Published:** 2016-12-09

**Authors:** Maho Kaminaga, Tadashi Ishida, Toru Omata

**Affiliations:** Department of Mechanical Engineering, School of Engineering, Tokyo Institute of Technology, Yokohama 226-8502, Kanagawa, Japan; ishida.t.ai@m.titech.ac.jp (T.I.); omata.t.aa@m.titech.ac.jp (T.O.)

**Keywords:** microfluidic device, pneumatic microvalve, inclined lithography, cell

## Abstract

We used inclined lithography to fabricate a pneumatic microvalve for tall microchannels such as those used to convey large cells. The pneumatic microvalve consists of three layers. The upper layer is the actual liquid microchannel, which has a parallelogram-shaped cross section of width 500 μm, height 100 μm, and an acute angle of 53.6°. The lower layer is a pneumatic microchannel that functions as an actuator, and the middle layer is a thin polydimethylsiloxane membrane between the upper and lower layers. The operation of the pneumatic microchannel actuator causes the thin membrane to bend, resulting in the bending of the liquid microchannel and its closure. It was confirmed that the closure of the liquid microchannel completely stopped the flow of the HeLa cell suspension that was used to demonstrate the operation of the microvalve. The HeLa cells that passed through the microchannel were also observed to retain their proliferation and morphological properties.

## 1. Introduction

Numerous cellular studies that utilized microfluidic devices have been conducted over the last decade [1]. The findings have afforded a precise understanding of cell behavior that could not have been obtained by conventional in vitro experiments. Microfluidic techniques, especially those that utilize microvalves, are employed in the development of microfluidic devices, which enable precise fluid control [2]. For example, a microvalve facilitates precise volumetric control [3] and rapid mixing [4] of biochemical reagents. The combination enables the development of on-chip biochemical processing devices [5].

**Figure 1:**
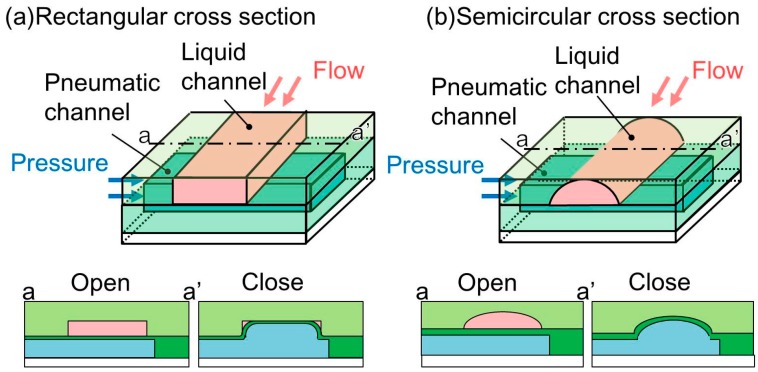
Conventional cross sections of microchannels of pneumatic pressure-driven microvalves. (**a**) Rectangular cross section; (**b**) Semicircular cross section.

The microvalves employed in microfluidic devices are classified based on their control and flow damming methods. The control methods include translation by electromagnetic actuators [6], variation of the fluid viscosity by an electric field [7], and displacement by pneumatic pressure [8]. Owing to their simplicity and compactness, pneumatic pressure-controlled microvalves are widely used. In the case of flow damming methods, examples include the deformation of the channel [8,9] and insertion of a partition [10]. Microvalves that utilize the deformation technique are quite common, primarily because of their simplicity and ease of fabrication. Hence, microvalves that utilize pneumatic pressure and channel deformation are often employed in microfluidic devices. A microvalve of this type is fabricated by placing a thin polydimethylsiloxane (PDMS) membrane between two microchannels [11]. Application of pneumatic pressure to one of the microchannels causes the thin membrane to bend, resulting in the other microchannel being closed [12]. However, such a microchannel with a rectangular cross section (Figure 1a), a replica of a photoresist mold, is incapable of completely shutting its corners to liquid flow. This is because of the inability of the membrane to fit into the right-angled corners of the microchannel. It may cause cross-contamination when microvalves are used for biological experiments with multiple cell types. Therefore, the leakage of the microvalves, even though it is very small, is not acceptable.

To solve this problem, a microchannel with a semicircular cross section has been proposed. This microchannel (Figure 1b), which is used to convey a liquid, has no corners and the membrane is able to fit securely against the wall, resulting in complete closure. The mold that is used to produce this type of microchannel is fabricated by a reflow process using a patterned photoresist. The patterned photoresist is melted at a high temperature and used to form a curved surface by the application of a surface tension. The height of the semicircular cross-sectional microchannel is commonly less than 30 μm [13]. To the best of our knowledge, we found only one situation where the semicircular cross-sectional microchannel of the pneumatic microvalve achieved 65 μm in height [14]. The process is thus not suitable for a pneumatic microvalve for a tall microchannel. The small cross-sectional area of a microchannel equipped with a microvalve produced by the reflow process cannot accommodate large cells such as egg and skeletal muscle cells. For these large cells, microvalves for tall microchannels, whose cross-sectional areas are large, have been developed. For example, a vertical membrane microvalve is fabricated using double-sided molding [15]. This microvalve could close a tall microchannel, though the three-dimensional channel configurations are complex. A microvalve for a tall semicircular cross-sectional microchannel is fabricated by grayscale lithography of SU-8 (thick negative photoresist) [16]. However, the technique requires a digital micro-mirror device, which is expensive. A more simple and inexpensive technique is thus required.

In the present study, we used an easy method to fabricate a pneumatic microvalve that enables complete closure of a tall microchannel. A single inclined lithography process was used to achieve the adopted parallelogram-shaped cross section of the microchannelis used. The effectiveness of the developed microvalve was verified by applying it to a cellular process.

## 2. Operating Principal of Pneumatic Microvalve for Tall Microchannel

**Figure 2:**
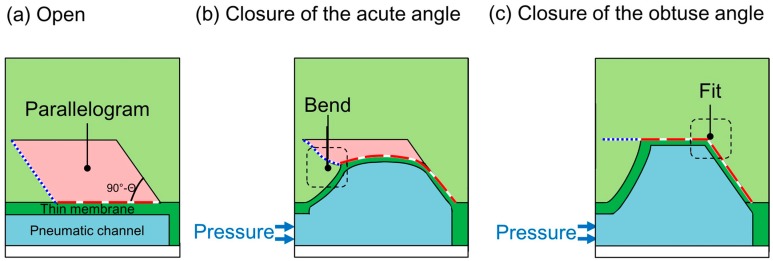
Operating principle of the proposed pneumatic microvalve for a tall microchannel. (**a**) Open; (**b**) Closure of the acute angle; (**c**) Closure of the obtuse angle.

**Figure 3:**
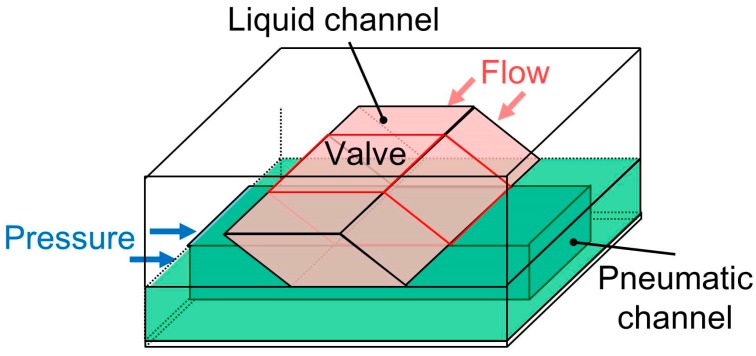
Constitution of the proposed pneumatic microvalve for a tall microchannel.

The operation of the proposed microvalve is based on the expansion of a pneumatic microchannel (Figure 2). The microvalve is composed of three layers. The first layer is the actual liquid channel, which has a parallelogram-shaped cross section. The second layer consists of a thin membrane in between the first layer and the third layer, which is the pneumatic microchannel (Figure 2a and Figure 3). The three layers are bonded, with the two microchannels orthogonally aligned to each other. When the pneumatic microchannel is pressurized, the thin membrane (red dash line) is bent and fits well against the wall of the liquid microchannel. An overhanging structure with a sharp edge at the acute-angled corner of the liquid microchannel (blue dot line) also bends to shut the corner (Figure 2b), enabling the membrane to easily fit into the obtuse-angled corner (Figure 2c). By this means, the liquid microchannel is completely shut.

This type of liquid microchannel with a parallelogram-shaped cross section can be made taller than one with a semicircular cross section because its fabrication does not involve the reflow process. The employed fabrication mold can be produced by inclined lithography [17] using thick-patterned photoresists. The achieved pneumatic microvalve is applicable to microfluidic devices equipped with tall microchannels, such as those used to handle large cells. Further, the microvalve with a tall microchannel might collaterally reduce shear stress applied to the cells.

## 3. Fabrication of Pneumatic Microvalve for Tall Microchannel

**Figure 4:**
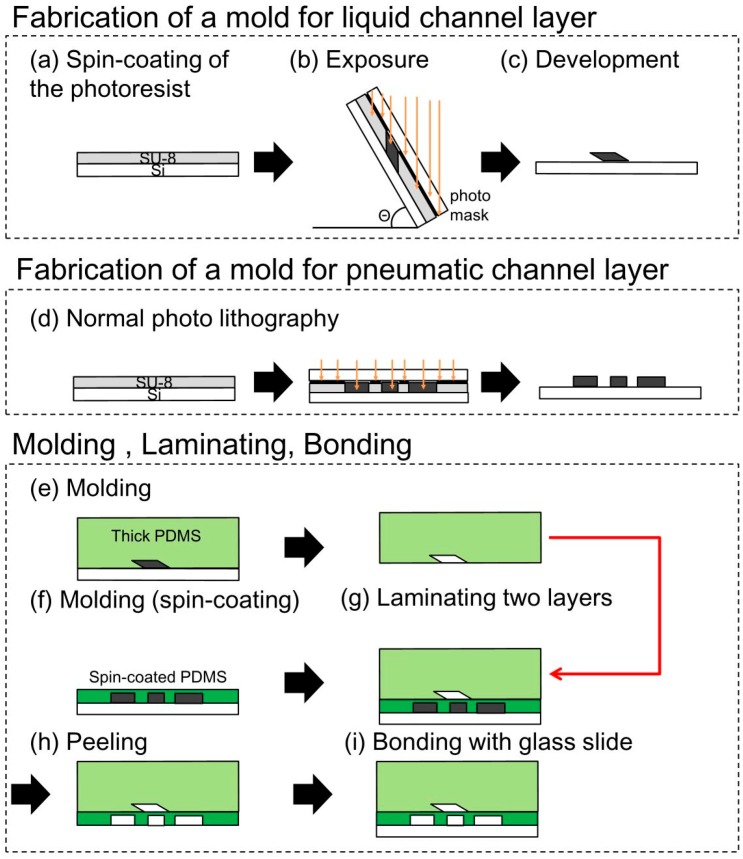
Fabrication process of the proposed pneumatic microvalve for a tall micro channel.

In the present study, the pneumatic microchannel was placed under the liquid microchannel. Both microchannels were fabricated by casting PDMS on molds produced by photolithography (Figure 4). The mold for the liquid microchannel, which had a parallelogram-shaped cross section, was produced by inclined lithography. The complete fabrication process was as follows: (a) SU-8 (SU-8 3025, Microchem, Westborough, MA, USA) of 50 or 100 μm in thickness was spin-coated on a Si substrate; (b) A photomask was aligned with and fixed to the Si substrate. The mask and the Si substrate were subsequently inclined at an angle θ (0°, 30°, and 60° were respectively considered in our experiment) and exposed to ultra violet (UV) light; (c) The patterned SU-8 for the mold of the liquid microchannel was developed; (d) The mold for the pneumatic microchannel was fabricated by normal lithography; (e) PDMS (Silpot 184 W/C, Dow Corning Toray, Midland, MI, USA) was cast on the mold of the liquid microchannel fabricated in (c); (f) The thin membrane between the liquid and pneumatic microchannel was fabricated by PDMS spin-coating on the mold of the pneumatic microchannel. The membranes of 30, 40, 80 μm in thickness were cured on the hotplate of 100 °C; (g) Before completing the cure process of the spin-coated PDMS, the PDMS layer of the liquid microchannel was detached from the mold and was attached to the spin-coated PDMS with an orthogonal alignment of the liquid and pneumatic microchannels. The attached PDMS layers are baked on the hotplate of 100 °C for the good bond between the PDMS layers; (h) The bonded PDMS layers were then detached from the mold of the liquid microchannel; (i) The bonded PDMS layers were then bonded to a glass substrate using the irradiation of vacuum UV/O_3_ using excimer lamp (SUS713, Ushio Inc., Tokyo, Japan). The PDMS used in all processes was a mixture of an elastomer and a cross-linker with a weight ratio of 10:1.

**Figure 5:**
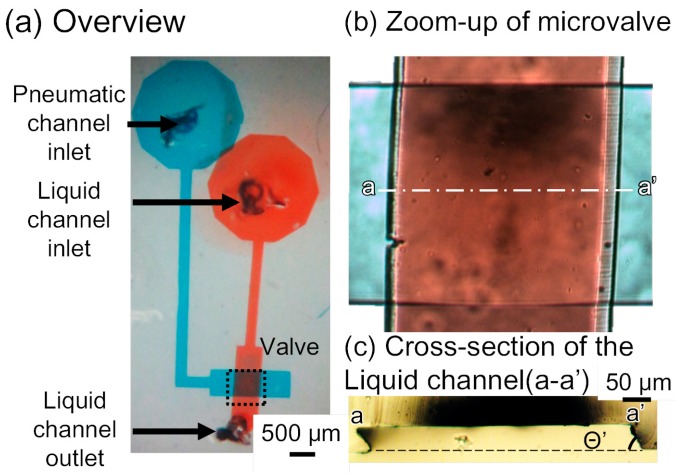
Microfluidic device with the microvalve for a tall microchannel. (**a**) Overview of the microfluidic device; (**b**) Zoom-up of the microvalve; (**c**) Cross-section of the liquid channel (a-a’).

Figure 5 shows the fabricated device. In the experiments performed in the present study, the liquid and pneumatic microchannels were respectively filled with red- and blue-dyed water to improve visibility. Figure 5b is the zoom-in image of an intersection of liquid and pneumatic microchannels. The intersection dimension is 500 μm in width (width of the liquid microchannel) and 500 μm in length (width of the pneumatic microchannel). Though we can scale down the dimensions, the mold of the liquid microchannel for a narrow intersection is difficult to fabricate due to a high aspect ratio. Moreover, the narrow pneumatic microchannel increases the response time of the microvalve. The both sides of the liquid microchannel are gradient in red, which shows the acute- and obtuse-angled structures. The cross section (a-a′ in Figure 5b) of the liquid channel before bonded to the pneumatic channel is shown in Figure 5c. The pneumatic and liquid microchannels both had a width of 500 μm and height of 50 μm. The acute angle of the parallelogram cross section of the liquid channel is measured to be 88.4°, 64.7°, and 53.6°, corresponding to incline angles θ of 0°, 30°, and 60° (90° − θ = 90°, 60°, and 30°), respectively. The difference is due to the refractive index of SU-8, which is 1.67 at the UV exposure wavelength [18]. According to Snell’s law [19], the acute angle of the parallelogram cross section of the liquid channel is given by θ′ = 90° − arcsin(sinθ/1.67). Thus, the calculated acute angle θ′ is: 90°, 72.5°, and 58.8° when θ becomes 0°, 30°, and 60° respectively. The measured acute angles are in good agreement with those theoretically anticipated, considering the wavelength of UV light. The minimum acute angle in SU-8 microstructures during inclined UV exposure is calculated to be 53.2° (= 90° − arcsin(1/1.67)). The operable inclination angle for inclined lithography and the operable membrane thickness were 60° and 40 μm, respectively, as determined by the experiments described in Section 4.2, Section 4.3, Section 4.4 and Section 4.5.

## 4. Characteristics of Pneumatic Microvalve for Tall Microchannel

### 4.1. Experimental Setup

**Figure 6:**
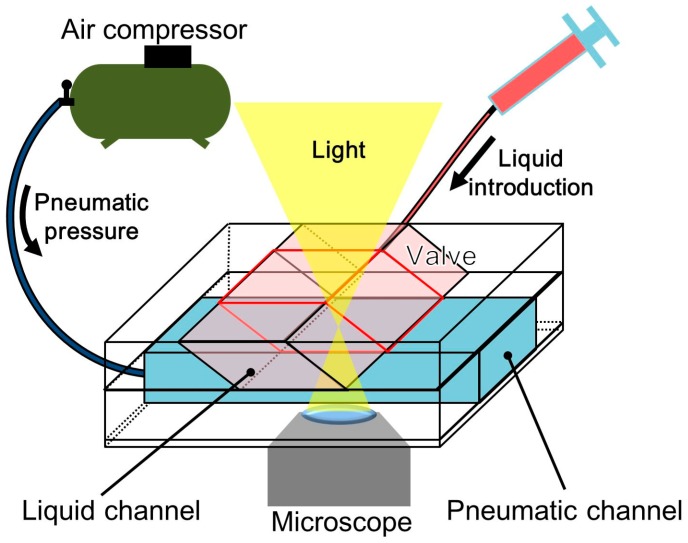
Experimental setup.

Figure 6 shows the experimental setup that was used to characterize the fabricated pneumatic microvalve. The microvalve was observed by a microscope (IX-73, Olympus, Tokyo, Japan). A liquid contained in a syringe connected to the liquid microchannel was introduced at a constant flow rate using a syringe pump (KDS200, KD Scientific, Holliston, MA, USA). Pressure was applied to the pneumatic microvalve using an air compressor (OFP-07005, Iwata, Kanagawa, Japan) and a pneumatic pressure regulator (IR1020-01BG-A, SMC, Tokyo, Japan). The compressed air flowed into the inlet of the pneumatic channel through an electromagnetic valve (SY114-5LZ, SMC), which was used as a pressure switch. All experiments were recorded using a National Television System Committee (NTSC) video camera (30 fps). Extracted frames from the videos were analyzed by using the ImageJ (National Institutes of Health (NIH), Bethesda, MD, USA) software to measure the brightness and the Tracker Video Analysis and Modeling Tool (Douglas Brown, Aptos, CA, USA) to track cell motion. Sometimes, the frames had afterimages of fast flowing cells. We did not analyze such fast flowing cells but relatively slow flowing cells with clear images.

### 4.2. Effect of Membrane Thickness on Microvalve Operation

**Figure 7:**
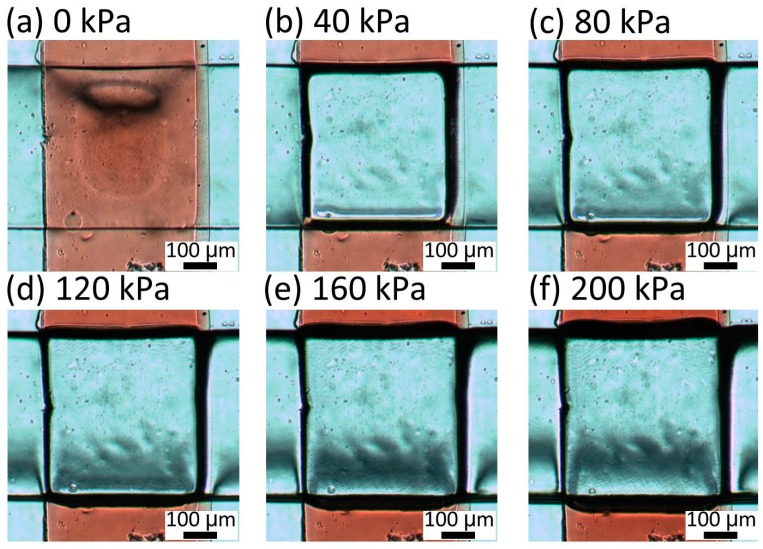
Center of intersection between the two microchannels of the microvalve with a 30-μm-thick membrane under pressures of (**a**) 0; (**b**) 40; (**c**) 80; (**d**) 120; (**e**) 160; and (**f**) 200 kPa.

**Figure 8:**
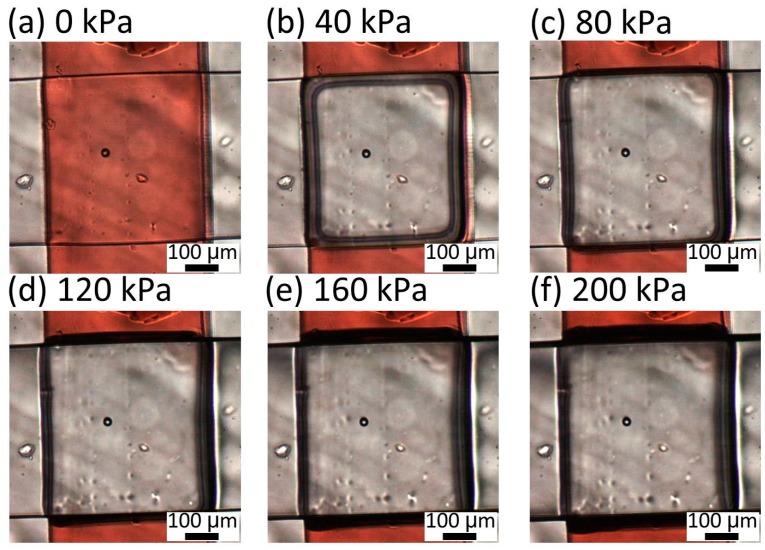
Center of intersection between the two microchannels of the microvalve with a 40-μm-thick membrane under pressures of (**a**) 0; (**b**) 40; (**c**) 80; (**d**) 120; (**e**) 160; and (**f**) 200 kPa.

**Figure 9:**
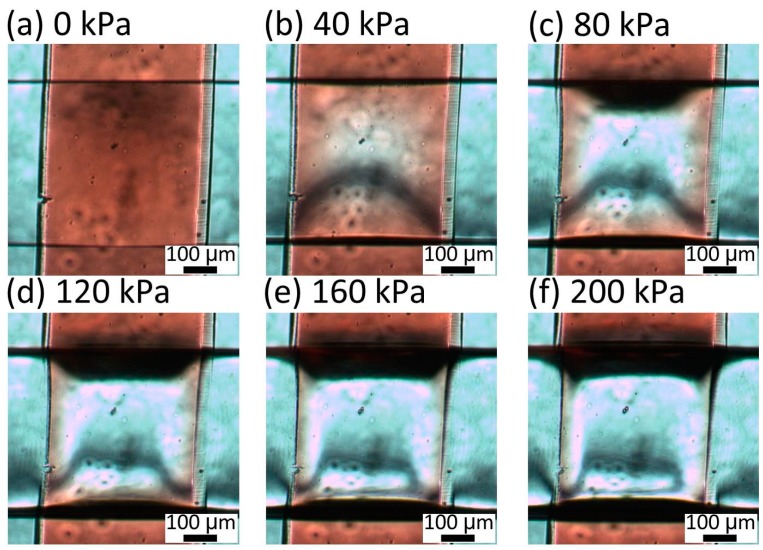
Center of intersection between the two microchannels of the microvalve with a 80-μm-thick membrane under pressures of (**a**) 0; (**b**) 40; (**c**) 80; (**d**) 120; (**e**) 160; and (**f**) 200 kPa.

To determine the appropriate thickness of the membrane between the liquid and pneumatic microchannels, the deformation of the membrane was observed for different thicknesses of 30, 40, and 80 μm, respectively. The liquid microchannel was filled with red-dyed water and pneumatic pressures between 0 and 200 kPa were applied in increments of 20 kPa. Figure 7 shows the microvalve with a 30-μm-thick membrane. When a pressure of 40 kPa was applied to this microvalve, the red area disappeared from the center of the intersection between the liquid and pneumatic microchannels. This indicates that the center of the intersection was completely shut. For applied pressures of 40–200 kPa, there was no change in the images of the microvalve, confirming that the center of the intersection was closed at 40 kPa and remained so for higher pressures. Figure 8 shows the microvalve with the 40-μm-thick membrane. In this case, the center of the intersection between the two microchannels was closed at 80 kPa. Figure 9 shows the microvalve with an 80-μm-thick membrane. The height at the center of intersection between the two microchannels proportionally decreased with increasing applied pressure, and the center of intersection was eventually closed at 120 kPa.

### 4.3. Estimation of Height of Liquid Channel during Valve Operation

**Figure 10:**
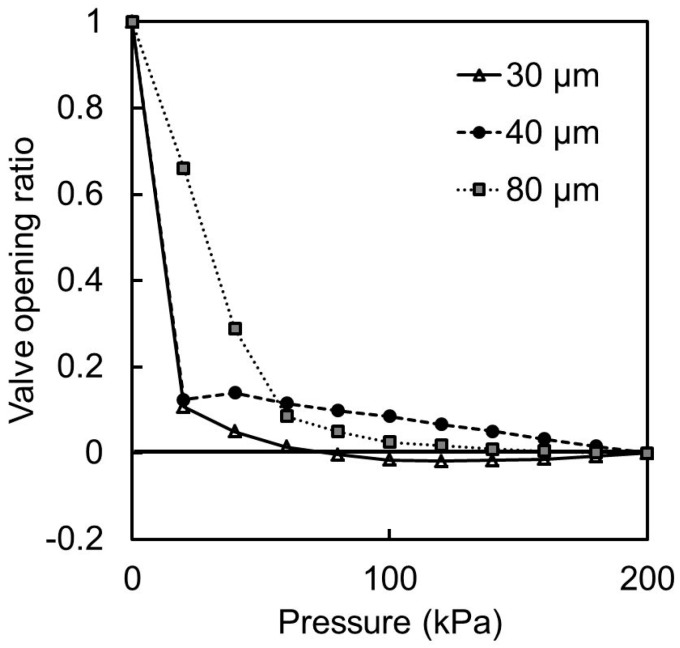
Normalized microvalve opening ratio as a function of the applied pressure for membrane thicknesses of 30, 40, and 80 μm.

The height of the liquid microchannel decreased when pressure was applied to the pneumatic microchannel. The thickness of the red-dyed water in the liquid microchannel also decreased with the height of the microchannel, and this caused the liquid to appear brighter. The height of the liquid microchannel could be estimated from the relationship between the brightness and the height. First, the brightness at the interconnection between the two microchannels without the application of a pressure was measured. The brightness under a pressure of 200 kPa with the valve closed was then also measured. The two brightness values were associated with liquid microchannel heights of 50 and 0 μm, respectively, and the linear correlation between the two parameters was derived. This was done for each membrane thickness. The correlation was used to estimate the height of the microvalve during the experiment. The microvalve opening ratio (the ratio of the height of the liquid channel during operation to the height when not operated) was adopted as an operation parameter and used to compare the different microvalves. Figure 10 shows the relationship between the applied pressure and the opening ratio of the microchannel.

**Figure 11:**
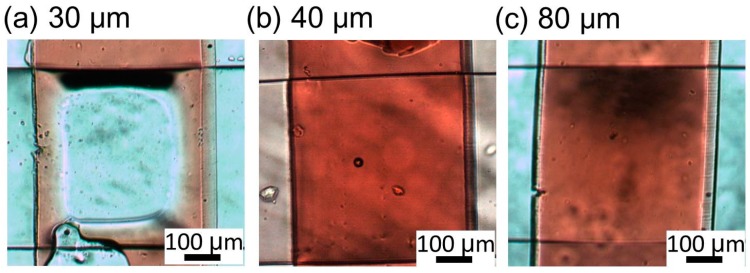
Reopened microvalves after pressure relief for membrane thicknesses of (**a**) 30; (**b**) 40; and (**c**) 80 μm.

From the experimental results, the microvalves with membrane thicknesses of 30 and 40 μm were determined to be most suitable for closing the center of the liquid microchannel. The microvalve opening ratio sometimes did not fall to zero because the reflection of light and shadow caused by the deformation of the membrane overlapped the measurement area and altered the brightness. Another important characteristic of the microvalve is its reopening when the pneumatic pressure is relieved. Figure 11 shows the microvalves after the pressure of 200 kPa was relieved. It can be seen that the microvalves with 40-μm- (Figure 11b) and 80-μm- (Figure 11c) thick membranes reopened after the relief of the pressure. This was not the case with the microvalve with the 30-μm-thick membrane (Figure 11a). This was because the thinness of the membrane caused the restoring force of the bent membrane to be smaller than the adhesive force between the membrane and the substrate.

The effect of the membrane thickness on the closure of the central part of the liquid microchannel was thus analyzed. However, the closure of the corners of the microchannel could not be evaluated by image analysis owing to the shadows formed at the edges. As described in the next section, the flow of a cell suspension through the liquid microchannel was used to investigate the closure of the corners.

### 4.4. Closure of Corners of Liquid Microchannel

To confirm the closure of the corners of the liquid microchannel, a cell suspension was passed into the microchannel during the operation of the microvalve. The microvalves with 40- and 80-μm-thick membranes were considered for this purpose. The flow rate of the cell suspension was 1 μL/min, and the pressure applied to the pneumatic microchannel was 200 kPa. The velocity of the cells through the liquid microchannel was measured to determine whether the microvalve completely closed the microchannel. The experiment utilized human cervical cancer HeLa cells obtained from ATCC (Manassas, VA, USA).

**Figure 12:**
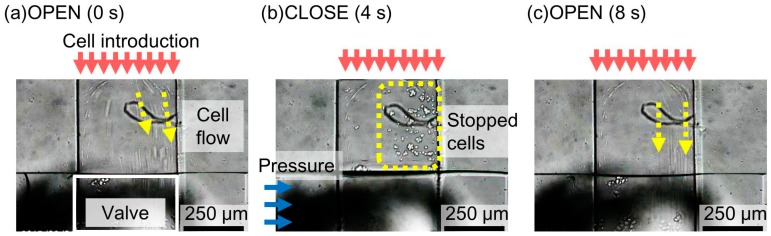
Damming of the cell suspension flow using the microvalve. (**a**) Cells in the cell suspension pass through the microvalve when the micro valve is open; (**b**) cells stops when the micro valve is closed; (**c**) cells flows againg when the micro valve is reopened.

**Figure 13:**
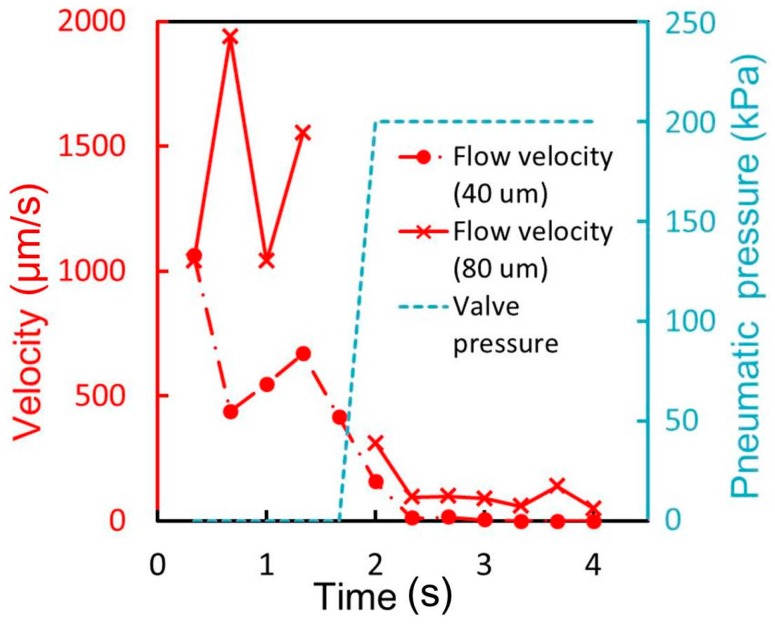
Time variations of the mean velocity of the flowing cells and the pressure applied to the microvalve for membrane thicknesses of 40 and 80 μm.

In the case of the microvalve with a 40-μm-thick membrane (Figure 12 and Video S1), the suspended cells were observed to move through the liquid microchannel when the microvalve was open (Figure 12a). However, the movement stopped when the microvalve was closed (Figure 12b). This indicated that the liquid microchannel including the corners was completely closed. The cells began to move again when the microvalve was reopened (Figure 12c). Figure 13 shows the time variations of the mean velocity of the flowing cells and the pressure applied to the pneumatic microchannel. The microvalve with a 40-μm-thick membrane is closed 0.35 s after the pressure of 200 kPa was applied. However, the microvalve with a membrane thickness of 80 μm could not completely stop the flow of the cells. The operable thickness of the membrane was thus determined to be 40 μm.

### 4.5. Effect of Incline Angle on Flow Damming

**Figure 14:**
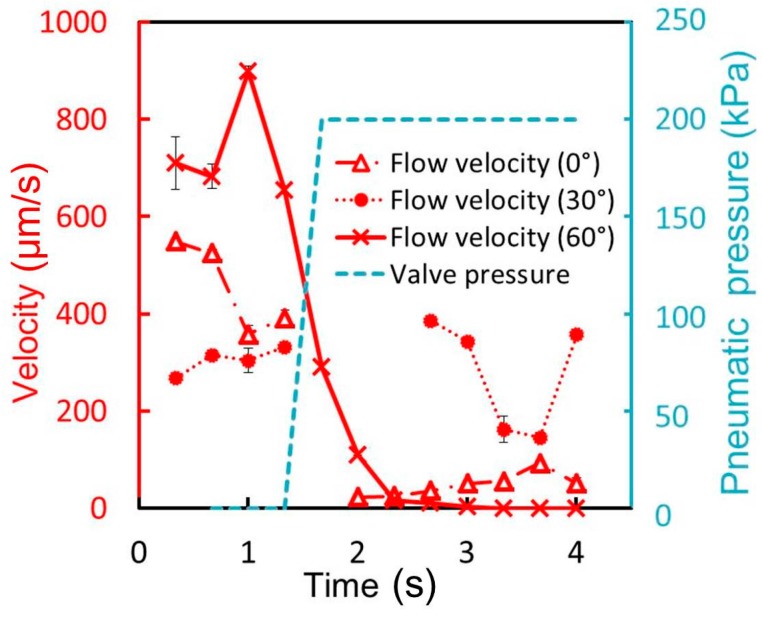
Time variations of the mean cell velocity and pressure applied to the pneumatic microvalve for θ = 0°, 30°, and 60° (*n* = 3).

The parallelogram cross-sectional valves can be assumed to be classified according to operating modes as follows: (A) the acute-angled structure is closed completely and the membrane fits well to the obtuse angle. Only in this case the liquid channel can be completely shut; (B) The acute-angled structure is not folded completely, but the membrane fits well to the obtuse angle; (C) The membrane does not fit well to the right angle. To determine the operable incline angle θ for a tall liquid microchannel, values of 0°, 30°, and 60° were respectively considered. The cell suspension was passed through the microchannel at 1 μL/min, and the pressure applied to the pneumatic microchannel was 200 kPa. The velocity of the cells was monitored to determine whether they stopped when the microvalve was operated. Figure 14 shows the time variation of the mean velocity of the flowing cells and the pressure applied to the pneumatic microchannel. In the case of the microchannel fabricated by UV lithography at 60° incline angle, the microvalve is closed 0.7 s after the pressure of 200 kPa was applied. However, in the case of the microchannels fabricated by UV lithography at 0° and 30° incline angles, the microvalve could not completely stop the flow of the cells. The results suggest that the case of a 60° incline angle corresponds to (A), that of 30° to (B) and that of 0° to (C). The operable incline angle for a tall liquid microchannel was determined to be 60°. We note that the cell flow was fast and afterimages of fast cells frequently appeared. Thus, we only measured the relatively slow flowing cells, and subsequently the standard deviation of the velocities of the cells appears to be small.

### 4.6. Effect of Liquid Mirochannel Height on Flow Damming

**Figure 15:**
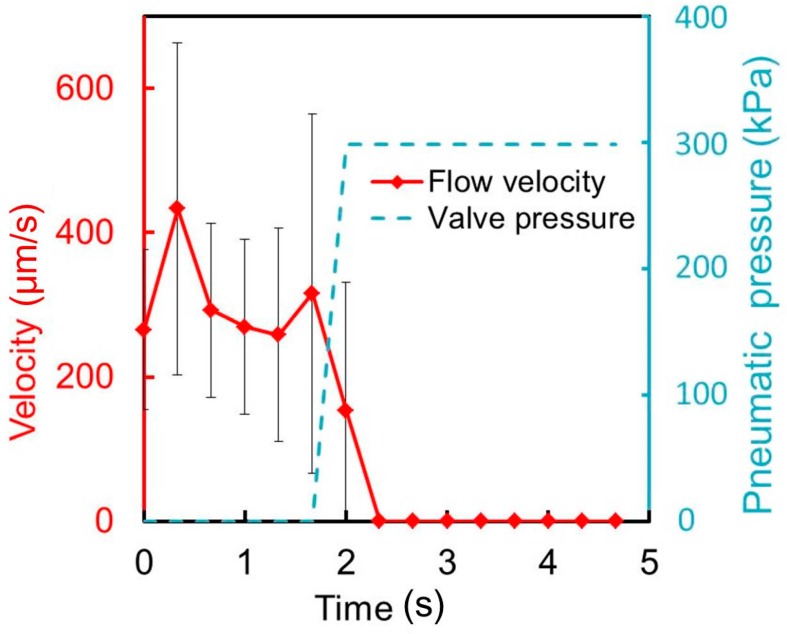
Time variations of the mean cell velocity and pressure applied to the pneumatic microchannel for the microvalve with a liquid microchannel height of 100 μm (*n* = 9).

A microvalve with a liquid microchannel of height 100 μm and θ = 60° was also fabricated and tested. Figure 15 and Video S2 shows the time variations of the mean velocity of the flowing cells and the pressure applied to the pneumatic microchannel for this microvalve. The flow rate of the supplied cell suspension during the test was 1 μL/min, and the pressure applied to the pneumatic channel was 300 kPa. Figure 15 shows the time variation of the mean velocity of the flowing cells and the pressure applied to the pneumatic microchannel. The movement of the cells was observed to stop 0.35 s after the pressure of 300 kPa was applied. The cross-sectional area of the liquid microchannel doubles, resulting in more slow flowing cells. Thus, the variation among the measured velocities was higher in that experiment. As a reference, a microvalve with an incline angle for a tall liquid microchannel θ = 0° was tested, and could not stop the flow of the cells (Video S3).

## 5. Demonstration of Use of Microvalve for Cellular Experiment

For its practical application, the microvalve was used to perform a cellular experiment in which a HeLa cell suspension was passed through the liquid microchannel of height 50 μm. The effect of the microvalve on the cells was examined in terms of the cell proliferation ratio and morphology as major factors of cellular status. The microvalve was randomly opened and closed 30 times over 10 min while the cell suspension, which contained 4.0 × 10^6^ cells/mL, was injected into the liquid microchannel. The cells were collected after passing through the microvalve and cultured in dishes. The cultured cells were counted and observed daily. As a control, cultured cells that had not been passed through the microvalve were counted and observed daily.

**Figure 16:**
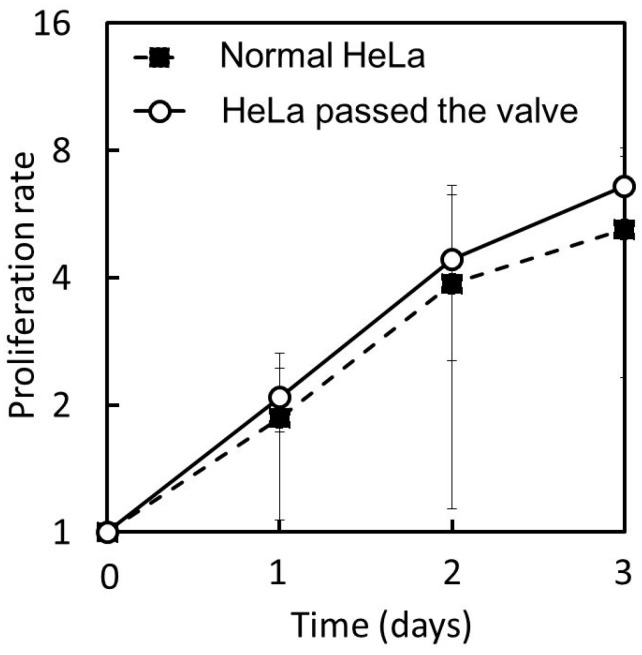
Proliferation curves of the HeLa cells passed and not passed (“Normal”) through the microvalve.

**Figure 17:**
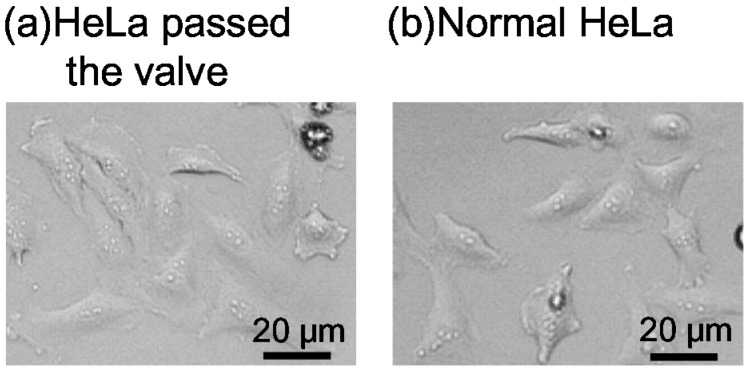
Day-3 morphologies of the HeLa cells (**a**) passed; and (**b**) not passed (“Normal”) through the microvalve.

Figure 16 shows the proliferation curves of the HeLa cells that were passed through the microvalve and those that were not passed. The cell proliferation rate was normalized by the initial number of cells and plotted on a binary logarithmic scale, considering that HeLa cells are known to be doubled daily. The proliferation curves for the two groups of cells can be observed to be approximately the same. Both cells approximately doubled in one day, and increased four-fold two days after cell seeding. The average proliferation ratio over three days of the cells passed through the microvalve was 6.6 (*n* = 3, S.D. = 1.15), while that of the cells that were not passed was 5.2 (*n* = 3, S.D. = 2.89). The cell morphologies for the two groups of cells were also examined, as shown in Figure 17a,b. No significant difference was observed. These observations suggest that the operation of the proposed microvalve did not largely affect the proliferation and morphologies in the HeLa cells.

## 6. Conclusions

We developed a pneumatic microvalve that can be used to control fluid flow through a tall microchannel for purposes such as the handling of large and stress-sensitive cells. HeLa cells were used in the demonstration of the proposed microvalve in this study. The movement of the cells through the tall liquid microchannel, which had a parallelogram-shaped cross section, was completely stopped when the microvalve was operated. The cells that passed through the microvalve were observed to exhibit similar proliferation and morphological properties as the original cells. We will investigate the influence of shear stress on stress-sensitive cells flowing through the microchannel in the future, and then integrate the microvalve into microfluidic devices for practical applications.

## Supplementary Materials

The following are available online at www.mdpi.com/2072-666X/7/12/224/s1; Video S1: Motion of the cell suspension passing though the microvalve with a liquid microchannel of height 50 μm, membrane thickness 40 μm and θ = 60°. Video S2: Motion of the cell suspension passing though the microvalve with a liquid microchannel of height 100 μm, membrane thickness 40 μm and θ = 60°. Video S3: Motion of the cell suspension passing though the microvalve with a liquid microchannel of height 100 μm, membrane thickness 40 μm and θ = 0°.
